# The Gupta Perioperative Risk for Myocardial Infarct or Cardiac Arrest (MICA) Calculator as an Intraoperative Neurologic Deficit Predictor in Carotid Endarterectomy

**DOI:** 10.3390/jcm11216367

**Published:** 2022-10-28

**Authors:** Juliana Pereira-Macedo, Beatriz Lopes-Fernandes, Luís Duarte-Gamas, António Pereira-Neves, Joana Mourão, Ahmed Khairy, José Paulo Andrade, Ana Marreiros, João Rocha-Neves

**Affiliations:** 1 Department of General Surgery, Hospital Centre of Médio Ave, 4760-124 Vila Nova de Famalicão, Portugal; 2 CINTESIS@RISE, Faculty of Medicine, University of Porto, 4200-450 Porto, Portugal; 3 Department of Angiology and Vascular Surgery, University Hospital Centre of São João, 4200-319 Porto, Portugal; 4 Faculty of Medicine and Biomedical Sciences, University of Algarve, ABC, Algarve Biomedical Centre, 8005-139 Faro, Portugal; 5 Department of Surgery and Physiology, Faculty of Medicine, University of Porto, Alameda Professor Hernâni Monteiro, 4200-319 Porto, Portugal; 6 Unit of Anatomy, Department of Biomedicine, Faculty of Medicine, University of Porto, Alameda Professor Hernâni Monteiro, 4200-319 Porto, Portugal; 7 Department of Anesthesiology, University Hospital Centre of São João, 4200-319 Porto, Portugal; 8 Departament of Anesthesiology, Faculty of Medicine, University of Porto, Alameda Professor Hernâni Monteiro, 4200-319 Porto, Portugal; 9 Department of Vascular and Endovascular Surgery, Assiut University Hospital, Assiut University, Assiut 71515, Egypt

**Keywords:** carotid endarterectomy, carotid stenosis, major adverse cardiovascular events, survival analysis, MICA score, atherosclerosis, perioperative stroke

## Abstract

Background: Patients undergoing carotid endarterectomy (CEA) may experiment intraoperative neurologic deficits (IND) during carotid cross-clamping. This work aimed to assess the impact of the Gupta Perioperative Myocardial Infarct or Cardiac Arrest (MICA) risk calculator in the IND. Methods: From January 2012 to April 2021, patients undergoing CEA with regional anaesthesia for carotid stenosis with IND and consecutively control operated patients without IND were selected. A regressive predictive model was created, and a receiver operating characteristic (ROC) curve was applied for comparison. A multivariable dependence analysis was conducted using a classification and regression tree (CRT) algorithm. Results: A total of 97 out of 194 included patients developed IND. Obesity showed aOR = 4.01 (95% CI: 1.66–9.67) and MICA score aOR = 1.21 (1.03–1.43). Higher contralateral stenosis showed aOR = 1.29 (1.08–1.53). The AUROC curve was 0.656. The CRT algorithm differentiated obese patients with a MICA score ≥ 8. Regarding non-obese patients, the model identified the presence of contralateral stenosis ≥ 55% with a MICA ≥ 10. Conclusion: MICA score might play an additional role in stratifying patients for IND in CEA. Obesity was determined as the best discrimination factor, followed by a score ≥ 8. A higher ipsilateral stenosis degree is suggested to have a part in avoiding procedure-related IND. Larger studies might validate the benefit of MICA score regarding the risk of IND.

## 1. Introduction

Carotid endarterectomy (CEA) is the standard recommended surgical technique for the treatment of asymptomatic patients but especially symptomatic patients with carotid stenosis [1,2]. A carotid stenosis of ≥50% is present in 1.5% of the population, mostly in men, representing roughly 58 million individuals worldwide [3].

The main concerns associated with this technique include perioperative embolic [4] and cerebrovascular events and myocardial infarction, which affect approximately 4% and <1% of patients, respectively [5,6,7]. 

This procedure may be executed under regional anaesthesia (RA) and requires carotid cross-clamping (CACC) of the affected carotid [4], during which a small proportion of patients manifest intraoperative neurologic deficits (IND) resulting from cerebral hypoperfusion [8]. Furthermore, the occurrence of IND has been shown to be a predictor of perioperative stroke [8].

The current literature is still unclear regarding the predictive factors of clamping intolerance. Previously, patients that manifested IND were found to have significantly higher decrement in mean flow velocity in the ipsilateral middle cerebral artery; two or more obstructions; and a reduced number of recruitable collateral vasculatures, assessed by transcranial doppler or magnetic resonance angiography (MRA) [9,10]. Recently, the use of both MRA and MRA perfusion with acetazolamide was found to carry a high sensitivity (100%) and specificity (95%) value of IND by using a calculated cut-off point of 0.322 [11]. Obesity and a higher degree of contralateral carotid stenosis were also described as independent predictors of IND [12].

The Gupta Perioperative Myocardial Infarct or Cardiac Arrest (MICA) calculator, derived from the National Surgical Quality Improvement Program (NSQIP), is a model widely used to assess the perioperative risk of intraoperative or postoperative myocardial infarction and cardiac arrest after noncardiac surgeries. This calculator is based on the type of surgery, functional status, serum creatinine, American Society of Anesthesiologists’ (ASA) class, and increased age as variables [13]. The NSQIP MICA risk calculator is currently validated as a predictor of MICA in aortic or other noncardiac vascular surgeries [13] of stroke in noncardiac surgery [14], as well as an accurate discriminator of major adverse cardiovascular events (MACE) following orthopaedic surgery [15,16].

The main goal of this study was to evaluate the predictive capacity of the NSQIP MICA risk calculator on the development of intraoperative neurologic deficits (IND). In addition, this study aimed to assess the influence and interactions that other variables might carry in the occurrence of IND.

## 2. Material and Methods

### 2.1. Study Population

Patients submitted to CEA under RA between 1 January 2012 and 30 April 2021 in a tertiary referral centre presenting with IND were prospectively and consecutively included from a cohort database, and post hoc analysis was carried out. Patients in the control group were selected with a 1:1 ratio as the immediately consecutive patient submitted to CEA without the occurrence of IND.

Patients were evaluated before surgery by a vascular surgeon and an anaesthesiologist. When previously symptomatic, patients were also assessed by a neurologist. The neurologist assessed symptomatic patients to detect any persistent sequelae relevant for further comparison, which is in congruence with current management guidelines [2]. Carotid stenosis was detected and quantified by a duplex ultrasound exam or a computed tomography angiogram (NASCET), or an additional duplex ultrasound exam performed by a different independent operator. Participants were evaluated postoperatively with clinical examination and Doppler ultrasound at 30 to 90 days after discharge and at 12 months of follow-up. Other data, such as demography and comorbidities, were assessed through clinical registries. This study was reported according to STrengthening the Reporting of OBservational studies in Epidemiology (STROBE) and Transparent Reporting of a multivariable prediction model for Individual Prognosis Or Diagnosis (TRIPOD) guidelines [17,18]. This study was approved by the University’s Institutional Review Board (248-18) on 14 July 2018, and informed consent was waived by the Ethics Committee. The ongoing cohort was registered for patient enrolment at clinicaltrials.gov (NCTNCT04347785, accessed on 3 December 2021). This study did not involve the assignment of patients to treatment groups.

### 2.2. Definitions

Intraoperative neurologic deficits were defined as any persistent symptomatic change or sign at neurologic awake examination (speech, motor function, and consciousness) during carotid artery cross-clamping, after hemodynamic adjustment [19]. Neuromonitoring is performed systematically to detect any subtle alteration that might be associated to cerebral ischemia, such as confusion, abrupted decrease in consciousness, or restlessness/agitation. Examples of tasks may include personal or time and space orientation questions, counting backwards, facial mimics, squeezing objects or limb mobility, direct commands from a surgeon, and assessment of consciousness level [20,21]. Event adjudication was performed by the anaesthesia–surgical team.

Carotid stenosis was considered symptomatic or asymptomatic according to the clinical practice guidelines of the European Society of Vascular Surgery [2]. 

Chronic kidney disease (CKD) was defined as an established diagnosis of CKD or a basal creatinine ≥ 1.5 mg/dL.

### 2.3. Surgical Technique

The surgical technique consisted of CEA followed by patch angioplasty, eversion technique, or direct suture. The choice of which approach was dictated by the intraoperative finding and the operator discretion. The surgeon also decided on the use of a carotid shunt when in the presence of IND. The centre has a reported stroke rate in CEA of 2% and a combined stroke/death rate of 2% (>100 procedures per year) [22]. Additionally, the acceptable major adverse events rate after CEA is described as 3% for asymptomatic patients and 6% for symptomatic patients, according to NSQIP Registry Carotid Endarterectomy Scale [23]. The exclusion criteria for RA are synchronous cardiac surgery and patient’s a priori unwillingness to stay awake during the intervention. Intraoperative neurologic monitoring consisted of consecutive brief awake neurological examinations and surveillance every 3 min during CACC. The neurological examinations prevailed in the definition of IND events [24]. Cerebral oximeter (INVOS™) data were additionally collected. Regional anaesthesia was performed by an instituted protocol and was executed in supine position by an ipsilateral deep cervical block. A 22-gauge insulated needle was inserted under image guidance with a 90-degree angle in the majority of cases. A 4–5 mL of ropivacaine 0.5% was then administered per spinal level (C2–C4) in a total amount of 12–15 mL (deep cervical blockade), followed by/or 5 mL of ropivacaine 0.5% at the halfway posterior border of the sternocleidomastoid muscle (superficial blockade) [25].

### 2.4. Statistical Analysis

Statistical analysis was performed with SPSS (IBM Corp., released 2020. IBM SPSS Statistics for Windows, version 27.0, Armonk, NY, USA). For a normal continuous variable distribution, mean ± standard deviation (SD) and median (interquartile range (IQR) 25 percentile to 75 percentile) were assessed for description. For asymmetric distributions, median (IQR) calculation was preferred. Categorical variables were displayed as *n* (%) in terms of function of the dependent variable. Comparisons in univariate analysis were performed using χ^2^ (or Fisher’s tests, if needed) for categorical variables and Student’s *t*-test or Mann–Whitney U test for continuous variables. A *p*-value inferior to 0.05 was considered as statistically significant. 

NSQIP MICA risk was calculated in terms of odds of outcome, according to the available online calculator supported by the existing evidence [13,26].

For a validation study, limited evidence is available regarding the necessary sample to assess the performance of an existing model. However, current recommendations support the use of around 200 patients for validation studies [18]. 

The regressive predictive model was created by resorting to regression analysis and dimension reduction by the method of backward feature elimination. Variables with clinical relevance included in the multivariable analysis were associated with the group with IND in the univariate analysis with statistical significance *p* < 0.1. On the basis of clinical and MICA score predictors with statistical significance for symptomatic intraoperative cerebral hypoperfusion, two formulas were created and evaluated: a previously validated clinical predictive model [27] and a clinical plus MICA predictive model. A receiver operating characteristic (ROC) curve was applied for the comparison of both models. 

To understand how some independent variables interacted with the dependent variable (IND), a tree format multivariable dependence analysis was conducted using a classification and regression tree (CRT) algorithm. This model is an adaptation of the Chi-squared Automatic Interaction Detector (CHAID) algorithm [28]. This technique was used for its ability to hierarchically rank the independent variables selected by the multivariable regression as an independent risk factor for IND (those with a *p* < 0.1) in specific algorithmic conditions (parent node = 10 cases and child node = 5 cases, in this case) by its ability to differentiate the distribution of the dependent variable, meaning the occurrence of IND, relying on the multivariable optimal binning algorithmic perspective used by the software (SPSS 27.0). Its tree-like method of result display allows for the visualisation of the variables hierarchically by the importance, degree of influence in the behaviour of the dependent variable, occurrence of IND, and determination of the optimal cut-off points, which maximises its outcome differentiation.

## 3. Results

### 3.1. Demographic and Clinical Data

The study population (*n* = 194) was mostly male (79.4%), with an overall mean age of 70.2 ± 9.0 years, ranging from 45 to 89 years old. Both study groups included 97 patients. A shunt was placed in 23 patients of the IND group. All the patients were under single antiplatelet (183 patients) or dual antiplatelet therapy (11 patients). No statistically significant differences were found between the IND and the control groups regarding gender (75.2% vs. 83.5%, *p* = 0.089), age (71.4 ± 9.2 vs. 69.0 ± 8.6, *p* = 0.065), or presence of cardiovascular risk factors, except for body mass index >30 kg /m^2^, which was significantly more frequent in the individuals that developed IND (27.8% vs. 8.2%, *p* ≤ 0.001) (Table 1).

The degree of CEA-intervened stenosis and of contralateral stenosis were the only other two patient variables that were found to significantly differentiate the control group from the patients who suffered IND. The mean degree of contralateral stenosis was significantly higher in the case group (66.6 ± 21.7 vs. 59.6 ± 15.2, *p* = 0.010), and the degree of ipsilateral stenosis was significantly higher in the control group (83.6 ± 9.6 vs. 82.1 ± 11.1, *p* = 0.020) (Table 1).

**Figure 1:**
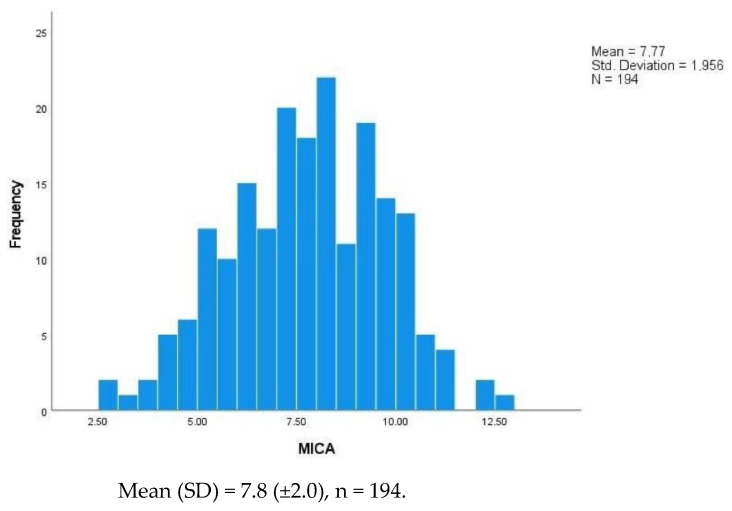
Histogram of the distribution of the Gupta Perioperative Risk for MICA Score. Abbreviations: MICA—Myocardial Infarction and Cardiac Arrest score; SD—Standard Deviation.

Concerning the population’s Gupta Perioperative Risk for MICA Score, it followed a normal distribution ranging from 2.7 to 12.8, with a mean score of 7.8 ± 2.0 (Figure 1, Table 1). Patients with IND showed a higher risk score, 8.1 ± 2.0, than patients in the control group, 7.5 ± 1.9 (*p* = 0.032).

### 3.2. Multivariable Analysis and Confounding Variables/Factors

Included variables in the regression model were age, sex, BMI > 30 kg/m^2^, ipsilateral stenosis, contralateral stenosis, and MICA score. After a multivariable regression analysis, only four of them were confirmed as independent factors for IND (Table 2). Obesity showed an aOR of 4.01 (95% CI 1.66–9.67), followed by a higher contralateral stenosis degree with an aOR of 0.69 (0.51–0.94) and MICA score with an aOR of 1.21 (1.03–1.43). Contrarily, a higher degree of ipsilateral stenosis was associated with an aOR of 0.69 (0.51–0.94).

### 3.3. The MICA Score Predictive Power

**Figure 2:**
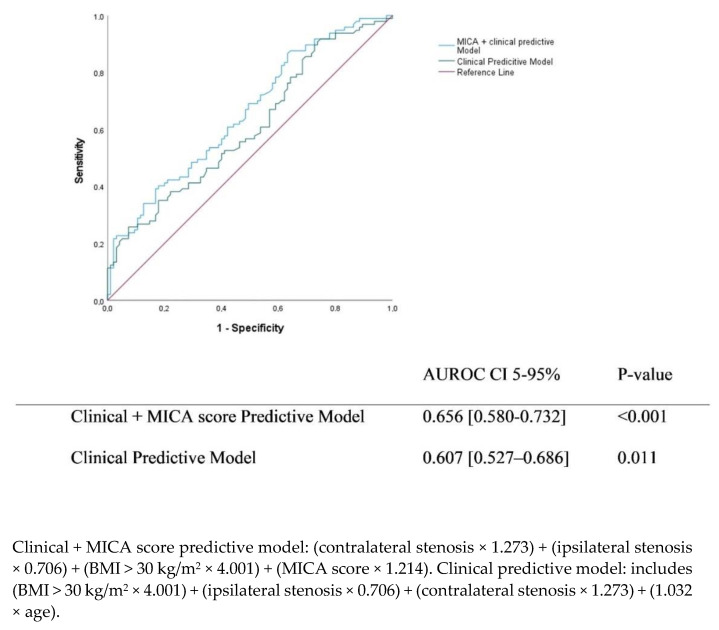
ROC curves of clinical and clinical plus MICA score models for symptomatic intraoperative neurological deficits. AUROC-area under the receiving operating characteristics; CI 5–95%: confidence interval 5–95%; MICA- Myocardial Infarction and Cardiac Arrest Score; Pseudo-R2 = 0.204-clinical plus MICA score predictive model; Pseudo-R2 = 0.188-clinical predictive model.

According to the calculated ROC curve analysis, the area was 0.656 (95% CI 0.580–0.732) when MICA was added to the clinical predicted model (composed of obesity and ipsilateral and contralateral stenosis degrees), which was higher (AUROC difference 0.049, *p* = 0.05), as observed in Figure 2. The optimal threshold obtained was 11.42, with a sensitivity of 87.6% and a specificity of 36.8%. Since the IND prevalence in the hospital centre is around 8%, the positive predictive value was 11%, and the NPV was 97%. 

**Figure 3:**
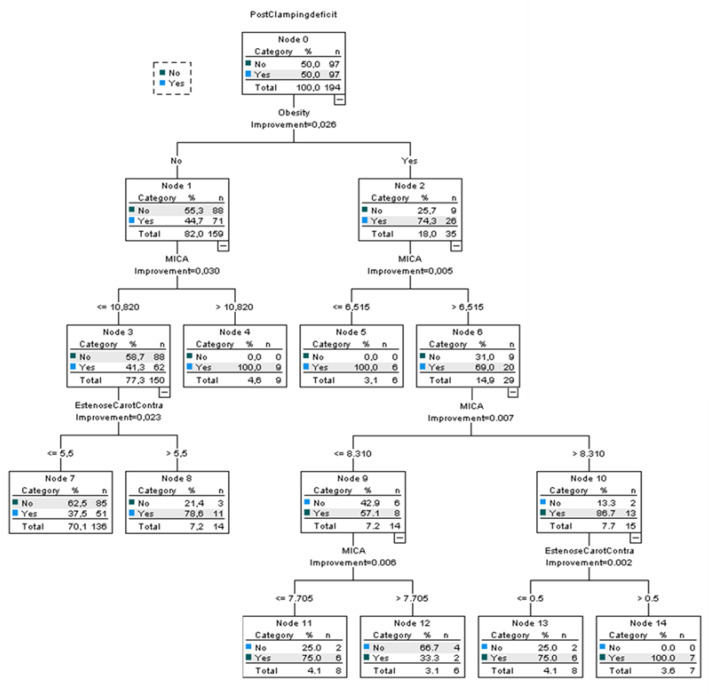
Classification and regression tree (CRT). The manifestation of IND was a dependent variable, while the presence or absence of a diagnosis of obesity, the degree of ipsilateral and contralateral stenosis, and the preoperative MICA score were the independent variables. Parent node = 10 cases; child node = 5 cases; maximum tree depth = 5; overall percentage = 69.1%.

Additionally, as shown in Figure 3, all obese patients with a preoperative MICA score > 8.310 and with contralateral carotid stenosis > 55% developed IND, as opposed to non-obese patients, who showed a higher preoperative MICA score cut-off point (>10.820) for the same outcome in the applied classification and regression tree. 

## 4. Discussion

The benefit of predicting the occurrence of IND during CACC in CEA relies on the fact that, although being a soft surgical outcome, it is interpreted as a sign of critical cerebral ischemia and it has already been shown to predispose to hard outcomes, such as stroke or other perioperative complications [8,29]. 

This study primarily demonstrates that a BMI > 30 kg/m^2^ is the main independent risk factor, increasing the risk of occurrence of IND in CEA by fourfold. Obesity has been associated with greater tortuosity of the extracranial internal carotid artery (OR 1.59; 95% CI, 1.35–1.86; *p* < 0.001), which may contribute to cerebral ischemia [30]. Increased risk for increased surgical time, cardiac complications, and death are also associated with obesity [31,32]. This risk is probably related to the potential these patients present for the necessity of an extensive surgical dissection and/or additional manipulation due to the higher adipose tissue content, predisposing to embolic events. The presence of obesity has also been shown to increase the risk for carotid plaque destabilisation, contributing to the occurrence of acute cerebrovascular symptoms, particularly in males aged < 70 years [33]. Factors such as a shorter neck may also contribute to a higher surgical intolerance [34]. 

A higher MICA risk score was demonstrated as being an independent factor for IND, with significant interaction with the remaining risk factors. In another retrospective study with 540,717 patients that underwent noncardiac surgery, MICA provided excellent results in predicting perioperative stroke, with a twofold increased risk for patients with higher scores and an overall AUC curve of 0.833 (0.825–0.842). Moreover, in this cohort, vascular surgery represented 12% of all noncardiac interventions [14]. However, MICA score was developed and validated to stratify patients according to their cardiac risk, with current evidence still lacking in terms of assessing the true impact of MICA on the occurrence of neurologic alterations [13]. Regarding the assessed AUC curve, a tendency was observed that significantly favours the applicability of adding the Gupta Perioperative MICA score over the clinical model. Age was not included individually in the ROC model because of collinearity with the MICA score, since the score already includes age itself. Furthermore, the value of the test is better perceived by its high NPV and low rate of false negatives, making it useful to rule out IND events. Still, the detected tendency may have relevance from a clinical point of view. Depending on the most frequent comorbidities or aetiology of the stenosis in the target population, it may be useful to consider the MICA score for the eligibility of the patients for CEA.

The study population also showed the presence of a higher degree of ipsilateral carotid artery stenosis as a protective factor for IND in CEA. This finding is probably explained by the increased compensatory collateral circulation from the contralateral carotid blood flow [35] and is documented in patients with unilateral carotid stenosis. This adaptation is absent in patients with bilateral stenosis, where posterior circulation is thought to compensate for the decreased brain perfusion [36]. This fact may also help to explain why a higher degree of contralateral stenosis is an independent risk factor for IND, as it compromises this adjustment. Another explanation consists in the presence of an ipsilateral hypoperfusion state in more than 80% of patients with carotid artery stenosis [37], which in chronic situations may result in the development of other adaptative mechanisms, leaving the patients with high-grade ipsilateral stenosis biologically better equipped to handle the ipsilateral CACC stress. The positive association between contralateral stenosis and the occurrence of IND has already been identified for other related outcomes, such as the increased risk of 30-day stroke/death, in-hospital strokes, and prolonged length of stay after CEA [27,38,39,40]. Nevertheless, it remains within the recommended guidelines, not being an absolute contraindication for surgery [27,38,39,40]. Having this in mind, both the presence and the degree of both ipsilateral and contralateral carotid artery stenosis are crucial during patient selection and surgical planning for CEA.

The female gender was also thought to display a higher rate of IND before the multivariable analyses but not afterward. A possible explanation may come from the low representativity of females in the sample, as a similar community-based study also showed conflicting results regarding this variable for other outcomes in CEA, with a crude incidence of indication for CEA that varied significantly by sex (female vs. male: HR 0.60 (95% CI 0.48–0.74)) but not after further adjustment for other confounding factors (female vs. male aHR 0.96 (0.76–1.22)) [41].

The use of the CRT technique allowed for the establishment of patients’ profiles on the basis of the differentiating power, at various levels/generations, of the independent variables, explaining the distribution of the occurrence of IND. This technique made it possible to determine MICA score cut-off points depending on the patient’s profile/generation in the CRT and added further validity to the logistic regression model. Considering this, the independent variable that primarily differentiates the occurrence or not of IND is obesity, with no influence of the independent variable contralateral carotid stenosis for non-obese individuals.

However, this work was not exempted from limitations, as this was a study with a sample size of under 200 individuals, mainly male, and from a single tertiary referral centre. Therefore, multicentre and larger sample studies are necessary to confirm and validate the present results. On the other hand, patients admitted to a tertiary referral centre are usually more endowed with significant comorbidities than those of peripheral centres, which may induce bias in interpreting results. Differences among surgical teams were also not assessed. Intraoperative hemodynamic parameters were not available and thus a correlation with the present results was not possible to establish.

## 5. Conclusions

MICA risk score might play some additional role in the preoperative period of CEA, stratifying patients for the occurrence of IND in CEA and consequently for worse surgical outcomes. The created predictive model with MICA has provided the ability to better rule out the event with a NPV of 97%. Additionally, obesity was shown to be the most dominant discriminating factor, followed by a MICA score above 8 points. For non-obese patients, a MICA score of 10 together with contralateral stenosis of at least 55% was also discriminative. A higher degree of ipsilateral stenosis is suggested to have a part in preventing procedure-related IND. 

However, conducting further studies in carotid surgery, with larger samples, is needed to assess the real benefit of the MICA score in predicting IND and stroke. Additionally, research on the risk score for ultra-selective prophylactic shunting or other strategies to prevent and reduce the development of IND is needed to improve perioperative outcomes.

## 6. Key Points

**Question:** What is the role of the National Surgical Quality Improvement Program (NSQIP) Gupta Perioperative Myocardial Infarct or Cardiac Arrest (MICA) risk calculator in predicting the risk of intraoperative neurologic deficits in patients undergoing carotid endarterectomy?
**Finding:** A negative predictive value of 97% was reached and a MICA score of at least 8, together with obesity, were shown to be the two greatest discrimination factors in patients that developed intraoperative neurologic deficits.
**Meaning:** MICA may have a role in stratifying patients according to their probability of developing intraoperative neurologic deficits.

## Figures and Tables

**Table 1:** General patient’s characteristics and preoperative comorbidities.

	Population *n* = 194	Controls *n* = 97	Cases of IND *n* = 97	*p*-Value
Gender				
Male, *n* (%)	154 (79.4)	81 (83.5)	73 (75.2)	0.089
Female, *n* (%)	40 (20.6)	16 (16.6)	24 (24.7)	
Age (years)				
Mean (±SD)	70.2 (±9.0)	69.0 (±8.6)	71.4 (±9.2)	0.065
Median (IQR)	71.5 (64–77)	71.0 (62.0–75.8)	72.0 (65.0–78.0)	
Side (right), *n* (%)	98 (50.5)	54 (55.7)	44 (45.3)	0.114
CV risk factors, *n* (%)				
Hypertension	171 (88.1)	85 (87.6)	86 (87.8)	0.865
Diabetes	82 (42.3)	38 (39.1)	44 (44.9)	0.454
Smoking history	100 (51.5)	55 (56.7)	45 (45.9)	0.113
Dyslipidaemia	166 (85.6)	82 (84.5)	84 (86.5)	0.953
BMI > 30 kg /m^2^	35 (18.0)	8 (8.2)	27 (27.8)	<0.001
CKD, *n* (%)	25 (12.9)	13 (13.4)	12 (12.3)	0.788
PAD, *n* (%)	47 (24.2)	25 (25.7)	22 (22.6)	0.559
CAD, *n* (%)	70 (36.1)	33 (34.0)	37 (38.1)	0.624
COPD, *n* (%)	24 (12.4)	14 (14.4)	10 (10.3)	0.354
CHF, *n* (%)	25 (12.9)	12 (12.3)	13 (13.4)	0.874
AF, *n* (%)	14 (7.3)	7 (7.2)	7 (7.2)	0.984
ASA, *n* (%)				
II	31 (16.0)	17 (17.5)	14 (14.4)	
III	150 (77.3)	74 (76.2)	76 (78.3)	0.610
IV	13 (6.7)	7 (7.2)	6 (6.1)	
Functional status, *n* (%)				
Independent	164 (84.5)	85 (87.6)	79 (1.4)	
Partially dependent	24 (12.4)	9 (9.3)	15 (15.4)	0.306
Dependent	6 (3.1)	4 (4.1)	2 (2.1)	
Asymptomatic, *n* (%)	106 (54.6)	53 (55.2)	53 (54.1)	0.875
Symptomatic, *n* (%)				
TIA	19 (9.8)	10 (10.3)	9 (9.3)	0.922
Stroke	69 (35.6)	33 (34.0)	36 (37.1)	
Stenosis degree				
Mean (±SD) (%)	83.8 ± 10.5	83.6 ± 9.6	82.1 ± 11.1	0.020
Median (IQR) (%)	80.0 (80–90)	90.0 (80–90)	70.0 (70–90)	
Contralateral stenosis degree				
Mean (±SD) (%)	63.1 ± 19.0	59.6 ± 15.2	66.6 ± 21.7	0.012
Median (IQR) (%)	50.0 (50.0–70.0)	50.0 (50.0–60.0)	60.0 (50.0–80.0)	
MICA				
Mean (±SD)	7.8 ± 2.0	7.5 ± 1.9	8.1 ± 2.0	0.032
Median (IQR)	7.8 (6.3–9.3)	7.9 (6.7–9.5)	7.7 (6.1–8.9)	

Abbreviations: AF—atrial fibrillation; ASA—American Society of Anesthesiologists Physical Status Classification System; CAD—coronary artery disease; CHF—cardiac heart failure; CKD—chronic kidney disease (creatinine ≥ 1.5 mg/dL); COPD—chronic obstructive pulmonary disease; CV—cardiovascular; IND—intraoperative neurologic deficits; IQR—interquartile range (25–75%); MICA—Gupta Perioperative Risk for Myocardial Infarction or Cardiac Arrest; obesity—body mass index > 30 kg/m^2^; PAD—peripheral artery disease; SD—standard deviation.

**Table 2:** Multivariable analysis by resorting to a regressive predictive model. Crude odds ratios (OR) and adjusted odds ratios (aOR) are displayed. The 95% confidence intervals (95% CIs) are indicated.

	Intraoperative Neurologic Deficit			
	Crude OR (95%CI)	*p*-Value	aOR (95%CI)	*p*-Value
Age	1.03 (0.99–1.06)	0.067	- *	-
Sex (female)	0.54 (0.27–1.10)	0.091	-	-
BMI > 30 kg/m^2^	4.18 (1.80–9.77)	0.000	4.01 (1.66–9.67)	0.002
Ipsilateral stenosis	0.72 (0.54–0.95)	0.022	0.69 (0.51–0.94)	0.019
Contralateral stenosis	1.22 (1.04–1.44)	0.014	1.29 (1.08–1.53)	0.004
MICA score	1.18 (1.01–1.36)	0.034	1.21 (1.03–1.43)	0.019

* Excluded also due to collinearity with MICA score. Abbreviations: aOR—adjusted odds ratio; BMI—body max index; MICA—Myocardial Infarction and Cardiac Arrest Score.

## Data Availability

The ongoing cohort was registered for patient enrolment at clinicaltrials.gov (NCTNCT04347785, accessed on 3 December 2021). This study did not involve assignment of patients to treatment groups.

## Abbreviations

MICA	Gupta Perioperative Risk for Myocardial Infarction or Cardiac Arrest
NSQIP	National Surgical Quality Improvement Program
ASA	American Society of Anesthesiologists
MACE	Major adverse cardiovascular event(s)
CEA	Carotid endarterectomy
IND	Intraoperative neurologic deficits
RA	Regional anaesthesia
MRA	Magnetic resonance angiography
CACC	Carotid artery cross-clamping
CKD	Chronic kidney disease
ROC	Receiver operating characteristic
AUROC	Area under the receiver operating characteristic curve
CRT	Classification and regression tree
SD	Standard deviation
IQR	Interquartile range
BMI	Body mass index
OR	Crude odds ratio(s)
aOR	Adjusted odds ratio(s)
CI	Confidence interval
NPV	Negative predictive value
STROBE	STrengthening the Reporting of OBservational studies in Epidemiology

## References

[1] Rerkasem A., Orrapin S., Howard D.P., Rerkasem K. (2020). Carotid endarterectomy for symptomatic carotid stenosis. Cochrane Database Syst. Rev..

[2] Naylor A.R., Ricco J.B., De Borst G.J., Debus S., De Haro J., Halliday A., Hamilton G., Kakisis J., Kakkos S., Lepidi S. (2018). Editor’s Choice—Management of Atherosclerotic Carotid and Vertebral Artery Disease: 2017 Clinical Practice Guidelines of the European Society for Vascular Surgery (ESVS). Eur. J. Vasc. Endovasc. Surg..

[3] Song P., Fang Z., Wang H., Cai Y., Rahimi K., Zhu Y., Fowkes F.G.R., I Fowkes F.J., Rudan I. (2020). Global and regional prevalence, burden, and risk factors for carotid atherosclerosis: A systematic review, meta-analysis, and modelling study. Lancet Glob. Health.

[4] Uno M., Takai H., Yagi K., Matsubara S. (2020). Surgical Technique for Carotid Endarterectomy: Current Methods and Problems. Neurol. Med. Chir..

[5] Howell S.J. (2007). Carotid endarterectomy. Br. J. Anaesth..

[6] Wu T., E Anderson N., Barber P.A. (2011). Neurological complications of carotid revascularisation. J. Neurol. Neurosurg. Psychiatry.

[7] Boulanger M., Camelière L., Felgueiras R., Berger L., Rerkasem K., Rothwell P.M., Touzé E. (2015). Periprocedural Myocardial Infarction After Carotid Endarterectomy and Stenting: Systematic Review and Meta-Analysis. Stroke.

[8] Pereira-Neves A., Fragão-Marques M., Rocha-Neves J., Gamas L., Oliveira-Pinto J., Cerqueira A., Andrade J., Fernando-Teixeira J. (2021). The Impact of Neutrophil-Tolymphocyte Ratio and Plateletto-Lymphocyte Ratio in Carotid Artery Disease. Port. J. Card. Thorac. Vasc. Surg..

[9] Anzola G., Limoni P., Cavrini G. (2008). Predictors of Carotid Clamping Intolerance during Endarterectomy That Would Be Wise to Apply to Stenting Procedures. Cerebrovasc. Dis..

[10] Montisci R., Sanfilippo R., Bura R., Branca C., Piga M., Saba L. (2013). Status of the Circle of Willis and Intolerance to Carotid Cross-clamping During Carotid Endarterectomy. Eur. J. Vasc. Endovasc. Surg..

[11] Myrcha P., Lewczuk A., Jakuciński M., Kozak M., Siemieniuk D., Różański D., Koziorowski D., Woźniak W. (2020). The Anatomy of the Circle of Willis Is Not a Strong Enough Predictive Factor for the Prognosis of Cross-Clamping Intolerance during Carotid Endarterectomy. J. Clin. Med..

[12] Rocha-Neves J., Pereira-Macedo J., Ferreira A., Dias-Neto M., Andrade J.P., Mansilha A.A. (2021). Impact of intraoperative neurologic deficits in carotid endarterectomy under regional anesthesia. Scand. Cardiovasc. J..

[13] Gupta P.K., Gupta H., Sundaram A., Kaushik M., Fang X., Miller W.J., Esterbrooks D.J., Hunter C.B., Pipinos I.I., Johanning J.M. (2011). Development and Validation of a Risk Calculator for Prediction of Cardiac Risk After Surgery. Circulation.

[14] Wilcox T., Smilowitz N.R., Xia Y., Berger J. (2019). Cardiovascular Risk Scores to Predict Perioperative Stroke in Noncardiac Surgery. Stroke.

[15] Peterson B., Ghahramani M., Harris S., Suchniak-Mussari K., Bedi G., Bulathsinghala C., Foy A. (2015). The Myocardial Infarction and Cardiac Arrest Risk Calculator is an Accurate Discriminator of Major Adverse Events Following Elective Hip and Knee Surgery. Circulation.

[16] Peterson B., Ghahramani M., Harris S., Suchniak-Mussari K., Bedi G., Bulathsinghala C., Foy A. (2016). Usefulness of the Myocardial Infarction and Cardiac Arrest Calculator as a Discriminator of Adverse Cardiac Events After Elective Hip and Knee Surgery. Am. J. Cardiol..

[17] Von Elm E., Altman D.G., Egger M., Pocock S.J., Gøtzsche P.C., Vandenbroucke J.P., Initiative S. (2014). The Strengthening the Reporting of Observational Studies in Epidemiology (STROBE) statement: Guidelines for reporting observational studies. Int. J. Surg..

[18] Moons K.G.M., Altman D.G., Reitsma J.B., Ioannidis J.P.A., Macaskill P., Steyerberg E.W., Vickers A.J., Ransohoff D.F., Collins G.S. (2015). Transparent Reporting of a multivariable prediction model for Individual Prognosis Or Diagnosis (TRIPOD): Explanation and Elaboration. Ann. Intern. Med..

[19] Stoneham M., Thompson J. (2009). Arterial pressure management and carotid endarterectomy. Br. J. Anaesth..

[20] So V.C., Poon C.C.M. (2016). Intraoperative neuromonitoring in major vascular surgery. Br. J. Anaesth..

[21] Rocha-Neves J.P., Pereira-Macedo J., Leite-Moreira A., Oliveira-Pinto J.P., Afonso G., Mourão J., Andrade J.P., Vaz R., Mansilha A. (2020). Efficacy of near-infrared spectroscopy cerebral oximetry on detection of critical cerebral perfusion during carotid endarterectomy under regional anesthesia. Vasa.

[22] Alves-Ferreira J., Rocha-Neves J., Dias-Neto M., Braga S.F. (2019). Poor long-term outcomes after carotid endarterectomy: A retrospective analysis of two portuguese centers. Scand. Cardiovasc. J..

[23] Dasenbrock H.H., Smith T.R., Gormley W.B., Castlen J.P., Patel N.J., Frerichs K.U., Aziz-Sultan M.A., Du R. (2019). Predictive Score of Adverse Events After Carotid Endarterectomy: The NSQIP Registry Carotid Endarterectomy Scale. J. Am. Heart Assoc..

[24] Carreira M., Duarte-Gamas L., Rocha-Neves J., Andrade J.P., Fernando-Teixeira J. (2020). Management of The Carotid Artery Stenosis in Asymptomatic Patients. Rev. Port. Cir. Cardiotorac. Vasc..

[25] Hakl M., Michalek P., Sevcik P., Pavlíková J., Stern M. (2007). Regional anaesthesia for carotid endarterectomy: An audit over 10 years. Br. J. Anaesth..

[26] Gupta P. (2022). Gupta Perioperative Risk for Myocardial Infarction or Cardiac Arrest (MICA)—MDCalc.: Mdcalc.com. https://www.mdcalc.com/calc/4038/gupta-perioperative-risk-myocardial-infarction-cardiac-arrest-mica#pearls-pitfalls.

[27] Pereira-Neves A., Rocha-Neves J., Fragão-Marques M., Duarte-Gamas L., Jácome F., Coelho A., Cerqueira A., Andrade J.P., Mansilha A. (2021). Red blood cell distribution width is associated with hypoperfusion in carotid endarterectomy under regional anesthesia. Surgery.

[28] Kass G.V. (1980). An Exploratory Technique for Investigating Large Quantities of Categorical Data. J. R. Stat. Soc..

[29] Piffaretti G., Tarallo A., Franchin M., Bacuzzi A., Rivolta N., Ferrario M., Ferraro S., Bossi M., Castelli P., Tozzi M. (2017). Outcome Analysis of Carotid Cross-Clamp Intolerance during Carotid Endarterectomy under Locoregional Anesthesia. Ann. Vasc. Surg..

[30] Wang H.-F., Wang D.-M., Wang J.-J., Wang L.-J., Lu J., Qi P., Hu S., Yang X.-M., Chen K.-P. (2017). Extracranial Internal Carotid Artery Tortuosity and Body Mass Index. Front. Neurol..

[31] Durup-Dickenson M., Nicolajsen C.W., Budtz-Lilly J., Laustsen J., Eldrup N. (2017). Body Mass Index and Operating Times in Vascular Procedures. EJVES Short Rep..

[32] Jackson R.S., Sidawy A.N., Amdur R.L., Macsata R.A. (2012). Obesity is an Independent Risk Factor for Death and Cardiac Complications after Carotid Endarterectomy. J. Am. Coll. Surg..

[33] Rovella V., Anemona L., Cardellini M., Scimeca M., Saggini A., Santeusanio G., Bonanno E., Montanaro M., Legramante I.M., Ippoliti A. (2018). The role of obesity in carotid plaque instability: Interaction with age, gender, and cardiovascular risk factors. Cardiovasc. Diabetol..

[34] Han T.S., Oh M.K., Kim S.M., Yang H.J., Lee B.S., Park S.Y., Lee W.J. (2015). Relationship between Neck Length, Sleep, and Cardiovascular Risk Factors. Korean J. Fam. Med..

[35] Henderson R.D., Eliasziw M., Fox A.J., Rothwell P.M., Barnett H.J.M. (2000). Angiographically Defined Collateral Circulation and Risk of Stroke in Patients With Severe Carotid Artery Stenosis. North American Symptomatic Carotid Endarterectomy Trial (NASCET) Group. Stroke.

[36] Fang H., Song B., Cheng B., Wong K.S., Xu Y.M., Ho S.S.Y., Chen X.Y. (2016). Compensatory patterns of collateral flow in stroke patients with unilateral and bilateral carotid stenosis. BMC Neurol..

[37] Khan A.A., Patel J., Desikan S., Chrencik M., Martinez-Delcid J., Caraballo B., Yokemick J., Gray V.L., Sorkin J.D., Cebral J. (2020). Asymptomatic carotid artery stenosis is associated with cerebral hypoperfusion. J. Vasc. Surg..

[38] Pothof A.B., Soden P.A., Fokkema M., Zettervall S.L., Deery S.E., Bodewes T.C., de Borst G.J., Schermerhorn M.L. (2017). The impact of contralateral carotid artery stenosis on outcomes after carotid endarterectomy. J. Vasc. Surg..

[39] Knappich C., Kuehnl A., Haller B., Salvermoser M., Algra A., Becquemin J.-P., Bonati L.H., Bulbulia R., Calvet D., Fraedrich G. (2019). Associations of Perioperative Variables With the 30-Day Risk of Stroke or Death in Carotid Endarterectomy for Symptomatic Carotid Stenosis. Stroke.

[40] de Borst G., Moll F., van de Pavoordt H., Mauser H., Kelder J., Ackerstaf R. (2001). Stroke from carotid endarterectomy: When and how to reduce perioperative stroke rate?. Eur. J. Vasc. Endovasc. Surg..

[41] Hicks C.W., Daya N.R., Black J.H., Matsushita K., Selvin E. (2020). Race and sex-based disparities associated with carotid endarterectomy in the Atherosclerosis Risk in Communities (ARIC) study. Atherosclerosis.

